# Nitrosopersulfide (SSNO^-^) targets soluble guanylyl cyclase and induces vasodilation in vivo

**DOI:** 10.1186/2050-6511-16-S1-A42

**Published:** 2015-09-02

**Authors:** Miriam M Cortese-Krott, Gunter GC Kuhnle, Alex Dyson, Bernadette O Fernandez, Marian Grman, Mark P Barrow, George McLeod, Karol Ondrias, Péter Nagy, Mervyn Singer, Malte Kelm, Anthony R Butler, Martin Feelisch

**Affiliations:** 1 Cardiovascular Research Laboratory, Department of Cardiology, Pneumology and Angiology, Medical Faculty, Heinrich Heine University of Düsseldorf, Universitätstrasse 1, 40225 Düsseldorf, Germany; 2 Department of Nutrition, University of Reading, Whiteknights, Reading RG6 6AP, UK; 3 Bloomsbury Institute of Intensive Care Medicine, University College London, Gower Street, London, WC1E 6BT, UK; 4 Clinical & Experimental Sciences, Faculty of Medicine, University of Southampton, Southampton General Hospital, Tremona Road, Southampton, SO16 6YD, UK; 5 Center for Molecular Medicine, Slovak Academy of Sciences, Vlarska 7; 83101 Bratislava, Slovakia; 6 Department of Chemistry, Warwick University, Coventry CV4 7AL, UK; 7 Bruker UK Ltd., Banner Lane, Coventry CV4 9GH, UK; 8 Department of Molecular Immunology and Toxicology, National Institute of Oncology, Ráth György utca 7-9, 1122, Budapest, Hungary; 9 Medical School, University of St-Andrews, St-Andrews, Fife, KY16 9AJ, Scotland, UK

## Background

Recent experimental evidence suggests that nitric oxide (NO) and hydrogen sulfide signaling pathways are intimately intertwined particularly in the vasculature, with mutual attenuation or potentiation of biological responses under control of the soluble guanylyl cyclase (sGC) / phopshodiesterase (PDE) pathway. There is now compelling evidence that part of the NO/sulfide cross talk has a chemical foundation via the formation of S/N-hybrid molecules including thionitrous acid (HSNO) and nitrosopersulfde (SSNO-). The aim of this study was to characterize the bioactive products of the interaction between sulfide and NO metabolites targeting sGC that may potentially regulate vasodilation.

## Results

We found that the chemical interaction of sulfide with NO or nitrosothiols leads to formation of S/N-hybrid metabolites including SSNO- via intermediate formation of HSNO. Contrary to a recent report in the literature but consistent with the transient nature of HSNO, its formation was not detectable by high-resolution mass spectrometry under physiologically relevant conditions. SSNO- is also formed in non-aqueous media by the reaction of nitrite with oxidized sulfur species including colloidal sulfur and polysulfides. SSNO- is stable in the presence of high concentrations of thiols, release NO, and activates sGC in RFL-6 cells in an NO-dependent fashion. Moreover, SSNO- is a potent vasodilator in aortic rings in vitro and lowers blood pressure in rats in vivo. The presence of high concentrations of SOD or thiols does not affect SSNO- mediated sGC activation, while it potentiates and inhibits the effects of the nitroxyl (HNO) donor Angeli's salt, suggesting that HNO release from SSNO- is not involved in sGC activation.

## Conclusion

The reaction between NO and sulfide leads to fomation of S/N-hybrid molecules including SSNO-, releasing NO, activating sGC and inducing vasodilation. SSNO- is considerably more stable than HSNO at pH 7.4 and thus a more likely biological mediator that can account for the chemical cross-talk between NO and sulfide.

